# Intestinal Bacteria Interplay With Bile and Cholesterol Metabolism: Implications on Host Physiology

**DOI:** 10.3389/fphys.2019.00185

**Published:** 2019-03-14

**Authors:** Natalia Molinero, Lorena Ruiz, Borja Sánchez, Abelardo Margolles, Susana Delgado

**Affiliations:** Department of Microbiology and Biochemistry of Dairy Products, Instituto de Productos Lácteos de Asturias – Consejo Superior de Investigaciones Científicas (IPLA-CSIC), Villaviciosa, Spain

**Keywords:** gut microbiota, bile acids, cholesterol, gut microbiota-host interplay, bile signaling

## Abstract

Bile is a biological fluid synthesized in the liver, mainly constituted by bile acids and cholesterol, which functions as a biological detergent that emulsifies and solubilizes lipids, thereby playing an essential role in fat digestion. Besides, bile acids are important signaling molecules that regulate key functions at intestinal and systemic levels in the human body, affecting glucose and lipid metabolism, and immune homeostasis. Apart from this, due to their amphipathic nature, bile acids are toxic for bacterial cells and, thus, exert a strong selective pressure on the microbial populations inhabiting the human gut, decisively shaping the microbial profiles of our gut microbiota, which has been recognized as a metabolic organ playing a pivotal role in host health. Remarkably, bacteria in our gut also display a range of enzymatic activities capable of acting on bile acids and, to a lesser extent, cholesterol. These activities can have a direct impact on host physiology as they influence the composition of the intestinal and circulating bile acid pool in the host, affecting bile homeostasis. Given that bile acids are important signaling molecules in the human body, changes in the microbiota-residing bile biotransformation ability can significantly impact host physiology and health status. Elucidating ways to fine-tune microbiota-bile acids-host interplay are promising strategies to act on bile and cholesterol-related disorders. This manuscript summarizes the current knowledge on bile and cholesterol metabolism by intestinal bacteria, as well as its influence on host physiology, identifying knowledge gaps and opportunities to guide further advances in the field.

## Introduction

The human gastrointestinal tract (GIT) is colonized by a vast array of microbes which dynamically interact with dietary and host-derived molecules in the intestinal lumen, significantly contributing to host physiology. Indeed, several animal and human studies have demonstrated that specific gut microbiota configurations contribute to inflammatory and metabolic diseases (Wu et al., 2015), although the precise molecular mechanisms behind the microbiota-host interactions impacting host health remain largely unknown. Cholesterol and bile acids (BAs) are important signaling molecules that, apart from exerting digestive functions, regulate multiple physiological processes in the host (Hegyi et al., 2018). Besides, the interaction of cholesterol and BAs with gut bacteria has been known for decades, although the role of these interactions in host health, and the possibility to modulate them through targeting the gut microbiota composition to improve human health, have only started to be recently explored.

Bile acids are synthesized in hepatocytes from cholesterol and conjugated to glycine and taurine before being secreted into the small intestine with the bile flow, which plays a major role in fat emulsification and absorption. Bile composition depends on the diet and intrinsic characteristics of the individuals, but usually contains over 50% BAs, over 20% fatty acids and cholesterol, and lower amounts of other molecules such as bilirubin or phospholipids (Farina et al., 2009). During its gastrointestinal transit, most BAs and cholesterol are reabsorbed in the distal small intestine, though a significant proportion evades this process, being excreted with feces (Islam et al., 2011).

Bile acids and cholesterol reaching the large intestine dynamically interact with our gut microbes. Indeed, BAs strongly compromise bacterial survival in the GIT, thus gut microbes must have developed mechanisms to counteract bile toxicity (Ruiz et al., 2013). Besides, gut microbial communities are capable of chemically modifying cholesterol and BAs, transformations that impact the gut microbiota and the BAs pool and, consequently, the signaling mechanisms they mediate. Accordingly, changes in this gut microbiota-bile axis are now acknowledged to have decisive implications in human health (Long et al., 2017).

The present minireview examines the current knowledge on the enzymatic activities of intestinal bacteria over BAs and cholesterol, and their implications in human physiology, with a particular emphasis on their impact on gastrointestinal disorders and aging-associated decline. Opportunities and limitations to translate this body of knowledge into novel microbiome-based applications for some of these diseases are also discussed.

## Cholesterol Metabolism by Intestinal Bacteria

Cholesterol is a terpenoid lipid with a carbon skeleton formed by four fused alicyclic rings. It is an essential component of the mammalian cell membranes and precursor of steroid hormones, vitamin D, and primary BAs (García et al., 2012). Following its GIT passage, most cholesterol is absorbed in the duodenum and proximal jejunum by a passive diffusion process. Reabsorbed cholesterol is incorporated with triglycerides and lipoproteins into transportable complexes called chylomicrons, which return to the liver through the enterohepatic circulation. The cholesterol escaping this re-absorption reaches the colon, where it can be metabolized by the intestinal microbiota and/or excreted with feces (Gérard, 2013).

**Figure 1:**
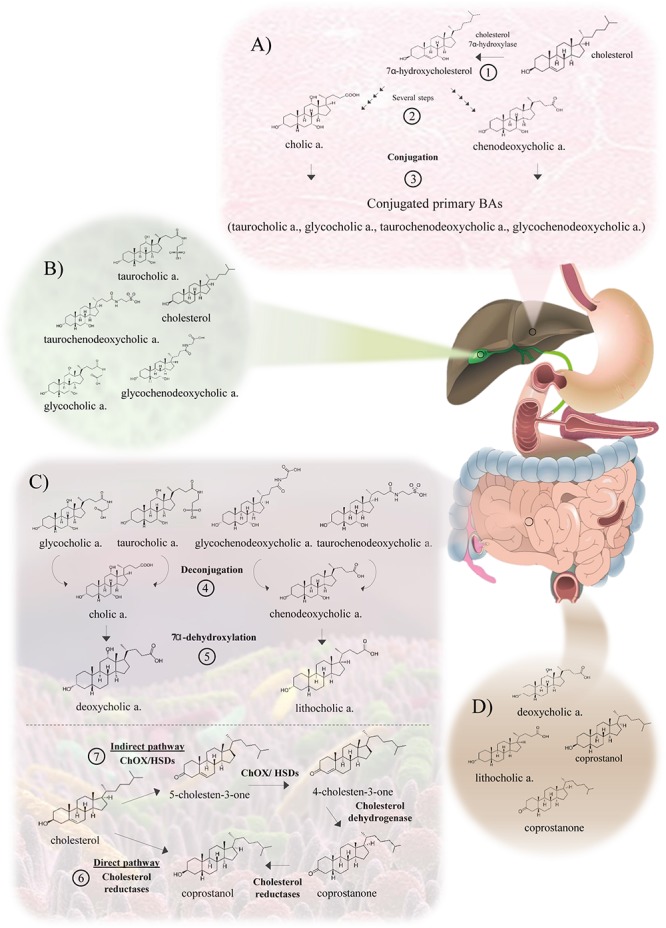
Bacterial cholesterol and bile metabolism in the gut, including microbiota-mediated transformations. **(A)** Metabolism of cholesterol in the hepatocyte. The conversion of cholesterol to primary BAs and their subsequent conjugation is carried out in the hepatocyte. (1) The primary BAs, cholic and chenodeoxycholic acids, are synthesized through the cytochrome P450 pathway. First, 7α-hydroxycholesterol is produced by the action of cholesterol 7α-hydroxylase. (2) Subsequently, several steps mediated by 12α-hydroxylase and 27α-hydroxylase generate the primary BAs. (3) The conjugation with glycine or taurine is mediated by the enzymes bile acid CoA synthetase and bile acid-CoA: amino acid N-acyltransferase. These conjugated BAs are excreted into bile by a BA export pump (BSEP) and stored in the gallbladder. **(B)** Bile composition. Conjugated primary BAs (glycocholic, taurocholic, glycochenodeoxycholic and taurochenodeoxycholic acids) are the main components of bile. Cholesterol, fatty acids, bilirubin and phospholipids are present in lower amounts. **(C)** Metabolism of BAs and cholesterol by intestinal bacteria. (4) The first reaction in the metabolism of BAs is the deconjugation or hydrolysis of conjugated BAs, catalyzed by bile salt hydrolases (BSHs). (5) Then, a bile salt 7α-dehydroxylase carries out the conversion of primary BAs to secondary BAs, deoxycholic and lithocholic acids. A part of the cholesterol is absorbed in the duodenum and proximal jejunum, returning to the liver. Remaining cholesterol reaches the large intestine, where it can be further metabolized by the intestinal microbiota or excreted with the feces. (6) Regarding cholesterol metabolism, the main gut microbial activity reaction involves the direct reduction of cholesterol to produce coprostanol, a reaction carried out by cholesterol reductases. (7) The indirect pathway begins with the oxidation of the 3β-hydroxy group by cholesterol oxidases (ChOx) or 3β-hydroxysteroid dehydrogenases/isomerases (HSD) to form 4-cholesten-3-one, and then cholesterol dehydrogenases produce coprostanone. Finally, cholesterol reductases form coprostanol. **(D)** BAs and sterols in feces. The main BAs in feces are secondary BAs, deoxycholic acid and lithocholic acid, with a lower concentration of primary BAs. Feces do also contain products of cholesterol metabolism such as coprostanol and coprostanone, that represent more than 50% of the total fecal sterols.

**Figure 2:**
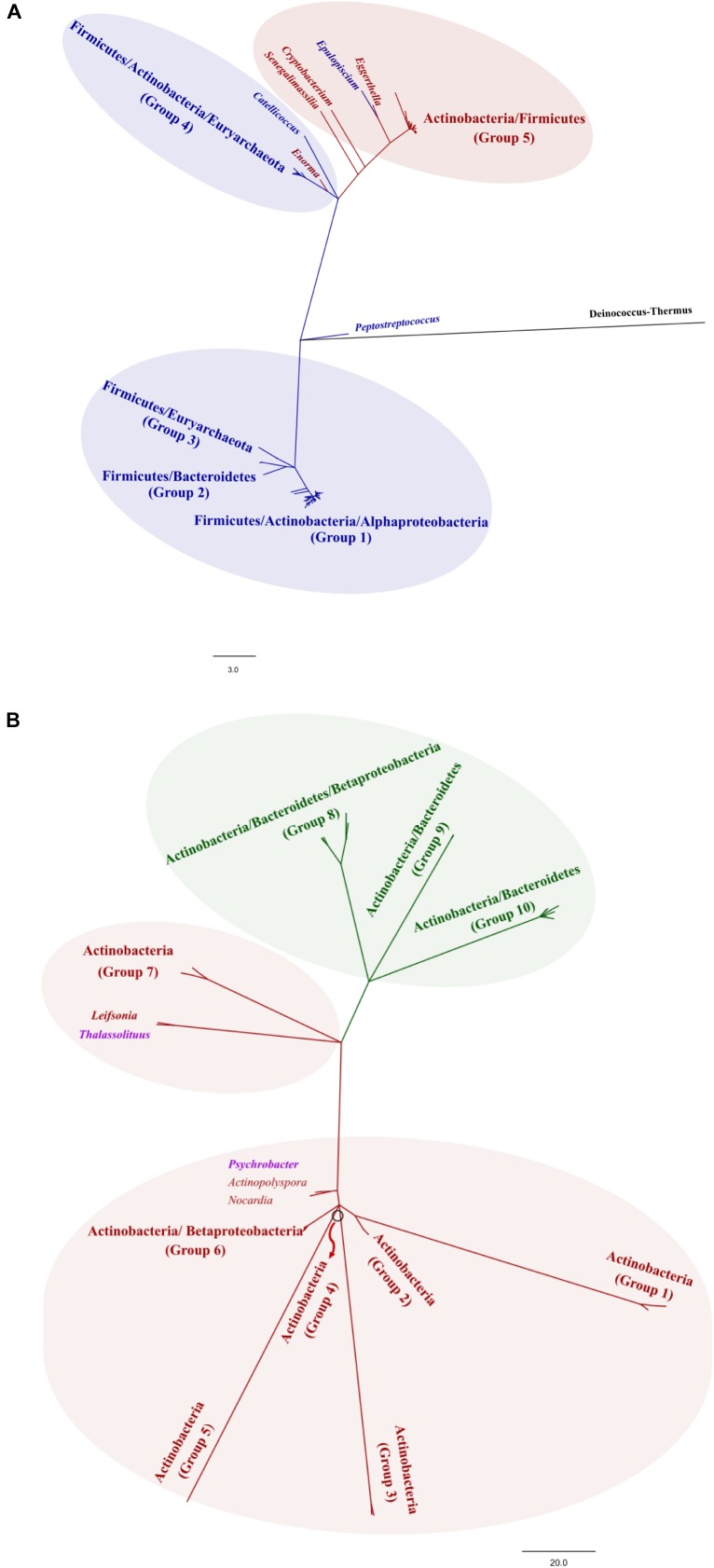
Phylogenetic analysis of bile salt hydrolases (BSH) **(A)** and cholesterol oxidases (ChOx) **(B)**. The construction of the phylogenetic trees and the clustering methods are described in detail in Supplementary Figure 1. The edition of the phylogenetic trees was performed with FigTree v1.3.1 (http://tree.bio.ed.ac.uk/software/figtree/). The trees were divided into groups, depending on the grouping at phylum level.

The metabolism of cholesterol by gut microbes has been described since the 30s (Schoenheimer, 1931) and has been supported by studies on germ-free animal models (Gérard et al., 2007). The microbial activities on cholesterol are based on its enzymatic reduction to produce coprostanone and coprostanol (Figure 1), which is poorly absorbable in the intestine. Thus, coprostanol production leads to increased cholesterol excretion into feces, contributing to reduce blood cholesterol level (Lye et al., 2010). Two different pathways have been proposed for this microbial reduction of cholesterol. The first pathway involves the direct reduction of the double bond 5–6 to give coprostanol, by cholesterol reductases (Gérard et al., 2004). The second pathway involves the oxidation of the 3β-hydroxy group and the isomerization of the double bond to produce 4-cholesten-3-one by cholesterol oxidases (ChOx) or 3β-hydroxysteroid dehydrogenases/isomerases (HSD) (García et al., 2012), followed by two reductions to form coprostanone and finally coprostanol (Gérard, 2013). Very limited information is available on the occurrence and distribution of the latter enzymes, although sequences belonging to ChOx are frequently found in the genomes of intestinal bacteria and gut/fecal metagenomes, indicating that cholesterol oxidation is a common activity in the gut microbiota. Remarkably, ChOx-encoding genes are found in the phyla Bacteroidetes, Proteobacteria and Actinobacteria, displaying a lower degree of conservation in Actinobacteria, but are absent in Firmicutes, one of the dominant phyla in the human gut microbiota (Figure 2 and Supplementary Figure 1).

Several factors throughout life, including changes in diet or antibiotics consumption (Korpela and Adlercreutz, 1985; Norin, 1997), have been suggested to affect the gut microbiota’s ability to reduce cholesterol to coprostanol, which exhibits higher rates of conversion in elderly individuals (Benno et al., 2009). Indeed, these factors are known to affect the gut microbiota composition in humans, although the real impact of lifestyle and other clinical factors in the microbial reduction of cholesterol, and the particular gut bacteria/activities implicated warrant further investigation.

Several cholesterol-reducing strains have been isolated from the intestine and feces of mammals (Eyssen et al., 1973; Brinkley et al., 1980, 1982). The first described cholesterol-reducing isolate of human origin was the *Bacteroides* sp. strain D8 (Gérard et al., 2007). Otherwise, only a few cholesterol-reducing intestinal bacteria have been identified, most of them belonging to the genus *Eubacterium*, although the genes or enzymes involved in this metabolism have not been well characterized yet.

Some other gut bacterial inhabitants, including lactobacilli and bifidobacteria species usually used as probiotics, have been long studied for their possible cholesterol-lowering activities. Although different mechanisms of action (involving removal, co-precipitation or assimilation) have been proposed (Pereira and Gibson, 2002; Liong and Shah, 2005; Tomaro-Duchesneau et al., 2014; Zanotti et al., 2015), to date, the real contribution of these microbial groups toward cholesterol-lowering and the molecular activities involved remain mostly unknown.

## Bacterial Bile Metabolism: Implications on Health and Disease

The metabolism of BAs by the gut microbiota has been known for decades, although its consequences on human health have only started to be considered (Farina et al., 2009; Islam et al., 2011; Gérard, 2013; Jia et al., 2017; Long et al., 2017), opening a new area of research in the microbiome-host interactions field. Key findings on this microbiota-BA signaling and host health are presented below.

### Metabolism of BAs by Intestinal Bacteria

The composition of the BAs pool in humans is determined by the enterohepatic cycle and the microbial metabolism of intestinal BAs. Briefly, the liver synthesizes two primary BAs from cholesterol, cholic acid and chenodeoxycholic acid, which are conjugated to either taurine or glycine before being poured into the bile flow. Conjugated BAs are the primary components of bile, which is stored in the gallbladder before being excreted into the small intestine during digestion. Over 95% of the BAs secreted in bile are reabsorbed in the terminal ileum, returning to the liver through the enterohepatic circulation, and only 5% reach the large intestine, being excreted in feces. In the large intestine, BAs can suffer several microbial-mediated transformations including deconjugation, carried out by bile salt hydrolases (BSHs) that hydrolyze the amide bond, and transformation of primary deconjugated BAs into secondary BAs mainly by a 7α-dehydroxylation (Figure 1). Whereas deconjugation reactions are carried out by a broad spectrum of colonic bacteria (Figure 2 and Supplementary Figure 1), 7α-dehydroxylation appears to be restricted to a limited number of intestinal bacteria (Ridlon et al., 2006). Thus, the BAs profile excreted in feces, mainly composed of secondary BAs, largely depends on the gut microbiota metabolism (Perwaiz et al., 2002).

#### Deconjugation of BAs

Bile salt hydrolases encoding genes have been detected and characterized in diverse gut microbes including species belonging to the genera *Bacteroides*, *Clostridium*, *Lactobacillus*, and *Bifidobacterium*, among others, being more diverse in members of the phylum Firmicutes (Figure 2 and Supplementary Figure 1) (Jones et al., 2008). BSH activity has been suggested as a BA detoxification mechanism for bacteria, although they may also obtain carbon, nitrogen and even sulfur from BA deconjugation. This latter element has relevance in the production of hydrogen sulfide that may have lasting health consequences as it increases colonocyte turnover and has been associated with inflammation and cancer (Carbonero et al., 2012). Through regulation of key genes involved in cholesterol metabolism and gastrointestinal homeostasis, BSH activity was proposed as a gut microbial activity with capacity to profoundly alter local (gastrointestinal) and systemic (hepatic) host functions as revealed by different studies in mice (Joyce et al., 2014).

#### 7-Dehydroxylation of BAs

The conversion of primary BAs to secondary BAs by 7α-dehydroxylases is probably one of the most physiologically relevant microbial transformations of BAs in humans (Duboc et al., 2013). Through 7α-dehydroxylation, the primary cholic acid is transformed into the secondary deoxycholic acid, and the primary chenodeoxycholic acid is transformed into the secondary lithocholic acid. To date, 7α-dehydroxylation activities have been characterized only in species belonging to the genera *Eubacterium* and *Clostridium*, including the species *Clostridium scindens* and *Clostridium hylemonae* (Ridlon et al., 2010). *C. scindens* is also capable of performing a 7β-dehydroxylation on ursodeoxycholic acid (the 7β epimer of chenodeoxycholic acid), yielding lithocholic acid (Ridlon et al., 2006, 2016).

#### Other Microbial Enzymatic Activities Acting on BAs

Other BA modifications such as amidation, oxidation-reduction, epimerization, esterification and desulfatation, can be carried out by intestinal microbes. Among them, oxidation-reduction and epimerization have received particular attention as some intestinal microbes synthesize HSD capable of performing reversible oxidation/reduction reactions and hydroxyl groups epimerization (Ridlon et al., 2016). Indeed, BA epimerization reactions have been largely overlooked due to the lack of appropriate analytical methods, although some iso-BAs have been suggested to represent the most abundant BAs in human feces (Hamilton et al., 2007). HSD activities are present in the four major phyla of the intestinal microbiota *Actinobacteria*, *Proteobacteria*, *Firmicutes*, and *Bacteroidetes* (Wahlströ et al., 2016), and the capability to carry out epimerization reactions has been characterized in several intestinal bacteria, including *Clostridium*, *Collinsella*, *Ruminococcus* or *Eubacterium* species (White et al., 1982; Lepercq et al., 2004; Liu et al., 2011; Lee et al., 2013). However, the physiological and functional significance of this metabolic activity remains largely unclear.

### Host Health Implications of Microbial Bile Metabolism

The microbial-mediated transformations of BAs at the intestinal level have been shown to be essential for intestinal and systemic health maintenance as the intestinal BAs and the gut microbiota mutually influence each other and, accordingly, BA-microbiota crosstalk disruption has been associated with several gastrointestinal, metabolic and inflammatory disorders, including those associated with aging-related decline (Jia et al., 2017), as summarized below.

#### BAs Metabolism and Inflammation

The gut microbiota-mediated biotransformation of the BA pool regulates BAs signaling by affecting the activation of host BA receptors such as the nuclear receptor farnesoid X receptor (FXR), which governs bile, glucose and lipid metabolism (Gadaleta et al., 2011). Indeed, a disrupted gut microbiota including reduced bile metabolizing bacteria significantly impairs BA metabolism and, consequently, the host metabolic pathways regulated by BA signaling, affecting glucose and cholesterol homeostasis, as well as immune states. Indeed, disorders associated with chronic low-grade inflammation have been linked to gut dysbiosis and altered BA profiles in humans (Chavez-Talavera et al., 2017), although few works have established a connection among specific activities of the microbiota on bile and cholesterol and the physiological alterations observed. As an example, analysis of existing gut metagenomic datasets evidenced that the abundance of the BSH gene *bsh* was significantly reduced in inflammatory bowel disease (IBD) and type-2 diabetes patients (Labbé et al., 2014). Accordingly, IBD patients evidenced increased fecal conjugated and sulphated BAs, and reduced fecal secondary BAs, suggesting the existence of characteristic alterations of bile metabolism associated with gut microbial shifts in IBD (Duboc et al., 2012, 2013). Indeed, some of these changes might be linked to dietary factors such as a diet high in saturated fat and increased sulfur-rich taurine conjugate BAs, which in turn promoted the expansion of the sulphite-reducing pathobiont *Bilophila wadsworthia* in mice. The resulting dysbiosis lead to an associated pro-inflammatory Th1 response and acute colitis in a mouse model, further demonstrating how microbial activity on a particular BA can impact inflammatory states and host health (Devkota et al., 2012).

#### BAs Metabolism and Colorectal Cancer

The relation between diet, microbial metabolism of BAs and human disorders, including colorectal cancer risk (CRC), is further supported by the fact that dietary fat increases biliary hepatic synthesis and, thus, the quantity of BAs that reach the colon, providing substrate for the synthesis of secondary BAs. These have been described as proinflammatory (Bernstein et al., 2011) and their increase may contribute to the pathogenesis of several gastrointestinal diseases, having been associated with colon polyps (de Kok et al., 1999) and CRC (Bernstein et al., 2005; O’Keefe et al., 2015). Indeed, fecal secondary BAs and microbial genes encoding for 7α-dehydroxylases were more common in African Americans who had a high risk of suffering CRC as compared with rural native Africans (Ou et al., 2013).

#### BAs Metabolism and Liver Diseases

Several chronic liver-related disorders, including non-alcoholic fatty liver disease (NAFLD), primary sclerosing cholangitis, steatosis and hepatic cancer – frequently associated with obesity – have been related to different intestinal microbial patterns (Adolph et al., 2018). In some of these diseases, an altered liver-microbiota-BAs crosstalk has also been defined. For instance, the ratio between primary and secondary BAs in feces and the levels of conjugated and unconjugated BAs in serum are higher in NAFLD patients (Kakiyama et al., 2013; Mouzaki et al., 2016; Jiao et al., 2018). Interestingly, an increase in taurine metabolizing activities has been evidenced in the gut microbiota of these patients, associated with increased representation of *Bilophila* species, and increased secondary BAs production (Jiao et al., 2018). Additionally, NAFLD is frequently associated with obese patients, for whom specific dysbiosis signatures have been defined (Gao et al., 2018). Consequently, in addition to affecting bile metabolism within the gut, the microbiota might also contribute to NAFLD pathogenesis through other mechanisms including increased energy intake, intestinal permeability and contribution to chronic pro-inflammatory states (Han et al., 2018), which go beyond the scope of this mini review.

#### Gut Microbiota Shifts in Aging Impact BAs Metabolism and Signaling

Gut microbiota changes throughout life, including loss of diversity, are associated with lifestyle and dietary changes in the elderly population, though they may also modulate elements of aging frailty such as innate immunity or cognitive function. Indeed, recent studies have evidenced that alterations in BAs metabolism accompany these aging-associated microbiota shifts and health decline. For instance, increased fecal excretion of deconjugated BAs has been observed in old mice in association with a shift toward pro-inflammatory states in the gut (Becker et al., 2019). In addition, a reduction in cholic acid and an increase in secondary BAs have been noticed in the serum of patients with Alzheimer disease (AD) (MahmoudianDehkordi et al., 2019), presumably reflecting augmented 7α-dehydroxylase activity in the gut microbiota. In fact, a mice model of AD has evidenced changes in the gut microbiota, including an increase in members of the *Clostridium* group, among which 7α-dehydroxylase activity is frequent (Brandscheid et al., 2017). Nevertheless, comprehensive studies of the gut microbiota and concomitant BAs metabolic changes in AD human cohorts are still lacking.

## Microbiota Modulation of Bile and Cholesterol Metabolism: Influence on Host Physiology and Signaling Mechanisms Involved

Several studies on germ-free animal models have evidenced the microbiota’s involvement in cholesterol and bile metabolism. For instance, the lack of gut microbiota in mice deficient in ApoE (a protein involved in the metabolism of fats) increased the plasma and liver cholesterol levels and reduced hepatic BAs synthesis (Kasahara et al., 2017). Also, the reverse cholesterol transport from peripheral tissues to the liver is augmented in germ-free mice (Mistry et al., 2017). These observations suggest that specific targeting of the intestinal microbiota could significantly impact cholesterol metabolism and cardiovascular diseases. Furthermore, germ-free animals lack secondary BAs production, and their microbial colonization modifies intestinal and serum BA fingerprinting, increasing total BAs concentrations (Joyce et al., 2014).

Since BAs are ligands of bile-responsive receptors involved in host metabolism, changes in BAs composition orchestrated by the intestinal microbiota activity, may affect their interaction with specific receptors, such as pregnane-activated receptor, vitamin D receptor, sphingosine-1-phosphate receptor, muscarinic receptor (Ridlon et al., 2016). Additionally, FXR, a nuclear transcription factor that regulates a wide range of genes (Teodoro et al., 2011), as well as the plasma membrane-bound G-protein coupled receptor TGR5 (Kawamata et al., 2003), have been remarkably characterized in relation to bile signaling. Both receptors are ubiquitously distributed in several tissues and have different affinity for individual BAs. TGR5 is mainly activated by the secondary BAs litocholic and deoxycholic acids, and recognizes both conjugated and deconjugated forms (Long et al., 2017). The most potent ligand for FXR is chenodeoxycholic acid, with cholic acid, deoxycholic acid and litocholic acid having a lower effect (Wahlströ et al., 2016). FXR activation can induce innate immune genes, promote the synthesis of antimicrobial agents acting on the gut microbiota (Inagaki et al., 2006), and regulate BA synthesis (Sinal et al., 2000). On the other hand, TGR5 plays a role in the regulation of BA and energy homeostasis (Wahlströ et al., 2016). Therefore, through these receptors, BAs act as signaling factors beyond the GIT. Further, considering that the gut microbiota deeply influences the BAs signature, different microbial communities can differentially impact bile signaling and determine the degree of activation of these receptors, with a concomitant impact on host metabolism. Indeed, BA receptors are currently considered therapeutic targets for several gastrointestinal and hepatic diseases (Firoucci et al., 2007); thus, microbiota-based approaches to modulate their activation may represent novel alternatives for certain disorders and warrant further investigation.

## Future Perspectives: Potential of Microbiota-Based Approaches to Modulate Bile Metabolism and Associated Conditions

In light of the recently unearthed gut microbiota-BA-host signaling interactions, microbiota-based approaches, from probiotics to dietary interventions, may become novel strategies to manage specific diseases linked to BAs metabolism dysregulation, as suggested by some *in vivo* studies (Devkota and Chang, 2015; Fukui, 2017). Most studies to date have focused on the potential of probiotics administration to reduce serum cholesterol levels. In this context, administration of probiotic strains to healthy mice increased deconjugation of BAs and fecal excretion (Jeun et al., 2010; Degirolamo et al., 2014) in association with increased BSH activity in the gut and overall modification of the microbiota composition (Degirolamo et al., 2014; Joyce et al., 2014; Tsai et al., 2014; Lye et al., 2017), changes that may have implications for host lipid metabolism. Indeed, a cholesterol-lowering effect was also observed following supplementation of a BSH-positive *Lactobacillus* strain to mice fed high-fat diets (Michael et al., 2017). However, limited studies have been conducted in human subjects in this regard. Remarkably, consumption of a BSH-positive *Lactobacillus* strain significantly reduced cholesterol in hypercholesterolemic subjects (Jones et al., 2012), although the observed effect might be the result of a complex metabolic re-arrangement, rather than solely a consequence of an increase in bile excretion.

Some probiotic interventions have also demonstrated their efficacy to ameliorate liver and inflammatory markers in models of NAFLD and IBD, although results are not yet conclusive (Han et al., 2018; Kobyliak et al., 2018). Besides, the strains tested in most studies were not specifically selected for their activities over bile metabolism, and the impact of the intervention on the fecal or serum BAs profiles, on the fecal microbiota composition or on their metabolic capability over bile and cholesterol, was not always evaluated. This strongly hampers establishing causal relationships between the metabolic activities of the microbiota over these compounds and the physiological effects observed.

Diet is another factor known to affect the gut microbiota and the BAs host signature. For instance, in a dietary intervention study in humans, a diet rich in animal-based fats was associated with increased excretion of secondary BAs, in accordance with an increased overall expression of *bsh* encoding genes in the gut microbiota, and an increase in the representation of potential pathobiont species such as *B. wadsworthia* (David et al., 2014). Thus, dietary strategies aimed at modulating BA metabolism through balancing the microbiota may represent alternative approaches to manage diseases linked to BA dysmetabolism (Ghaffarzadegan et al., 2018). Though these have been scarcely studied in humans, studies in mice models have showed the potential of certain dietary ingredients to modulate gut microbiota and BAs profile. For instance, *Akkermansia muciniphila* enrichment through administration of epigallocatechin-3-gallate prevented diet-induced obesity and regulated bile signaling (Sheng et al., 2018), although the contribution of changes in specific microbial metabolic activities over bile and cholesterol in this model has not been determined.

## Conclusion

In summary, it has become increasingly clear that BAs exert a much wider range of biological activities than initially recognized and that BAs, gut microbiota and health status are closely linked and hold a yet- underexplored valuable potential to design novel diagnostic and therapeutic approaches based on specific gut microbiota activities. Elucidating the molecular mechanisms underlying the gut microbiota-BA-host health interplay will establish the basis to fully understand the gut microbiota potential to modulate bile metabolism and host health. Further studies using specifically designed *in vivo* models or human trials, and exploiting microorganisms or activities with demonstrated capacity to specifically act on selected BAs, are necessary for aiding the development of novel microbiome-based approaches for disorders associated with BAs dysregulation.

## Author Contributions

AM, SD, and BS conceived and organized the manuscript. NM designed the figures. NM, LR, BS, AM, and SD contributed to the writing, critically reviewed the manuscript, and approved the final version of the manuscript.

## Conflict of Interest Statement

The authors declare that the research was conducted in the absence of any commercial or financial relationships that could be construed as a potential conflict of interest.
