# Supplementary material for: Intestinal Bacteria Interplay With Bile and Cholesterol Metabolism: Implications on Host Physiology
Source: Front Physiol. 2019 Mar 14;10:185. doi: 10.3389/fphys.2019.00185 (PMC6426790; doi:10.3389/fphys.2019.00185)
Supplement: Supplementary file 1 [file Image_1.pdf]

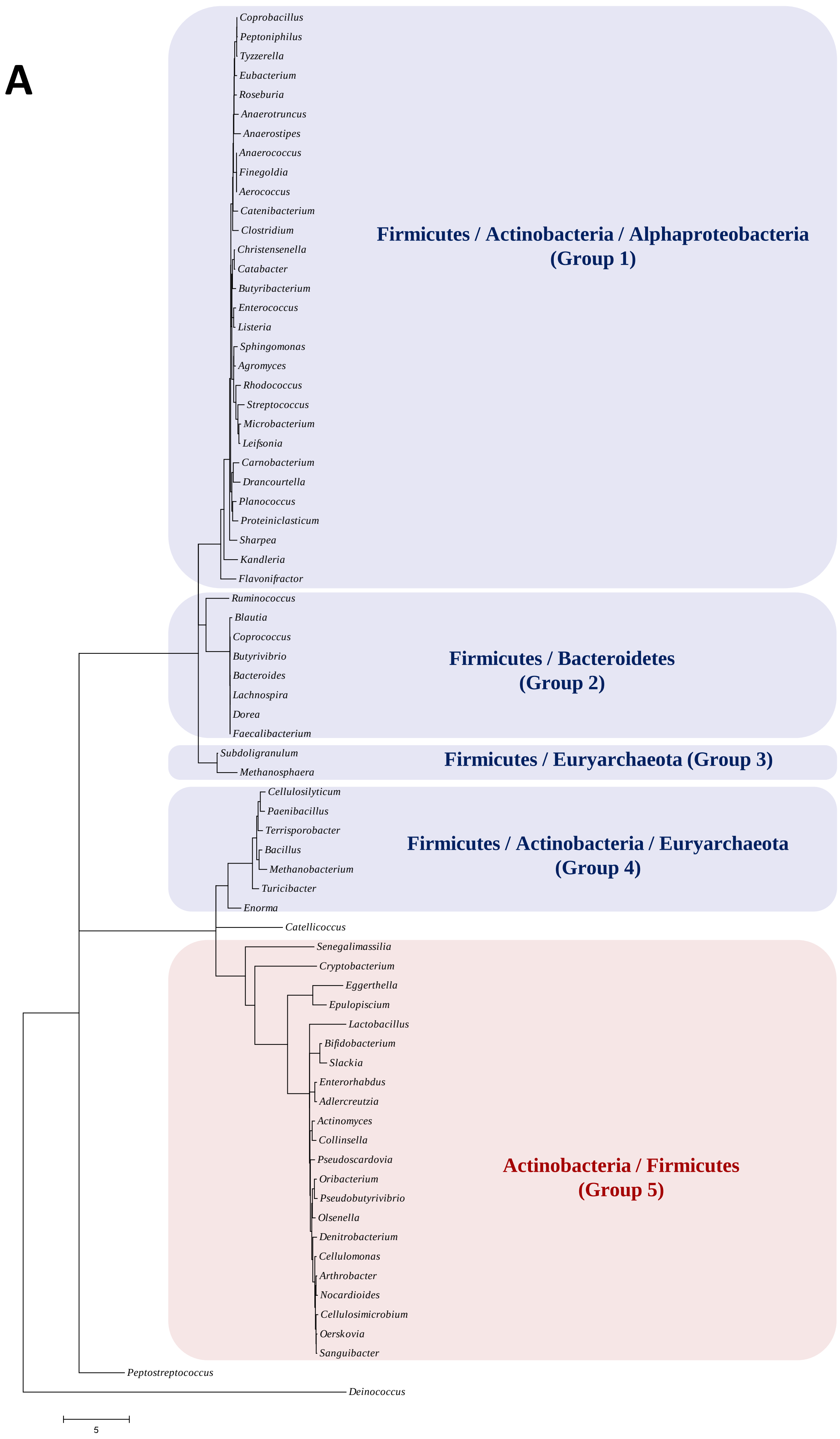
A
Coprobacillus
Peptoniphilus
Tyzzerella
Eubacterium
Roseburia
Anaerotruncus
Anaerostipes
Anaerococcus
Finegoldia
Aerococcus
Catenibacterium
Clostridium
Christensenella
Catabacter
Butyribacterium
Enterococcus
Listeria
Sphingomonas
Agromyces
Rhodococcus
Streptococcus
Microbacterium
Leifsonia
Carnobacterium
Drancourtella
Planococcus
Proteiniclasticum
Sharpea
Kandleria
Flavonifractor
Firmicutes / Actinobacteria / Alphaproteobacteria (Group 1)
Ruminococcus
Blautia
Coprococcus
Butyrivibrio
Bacteroides
Lachnospira
Dorea
Faecalibacterium
Firmicutes / Bacteroidetes (Group 2)
Subdoligranulum
Methanosphaera
Firmicutes / Euryarchaeota (Group 3)
Cellulosilyticum
Paenibacillus
Terrisporobacter
Bacillus
Methanobacterium
Turicibacter
Enorma
Firmicutes / Actinobacteria / Euryarchaeota (Group 4)
Catellicoccus
Senegalimassilia
Cryptobacterium
Eggerthella
Epulopiscium
Lactobacillus
Bifidobacterium
Slackia
Enterorhabdus
Adlercreutzia
Actinomyces
Collinsella
Pseudoscardovia
Oribacterium
Pseudobutyrivibrio
Olsenella
Denitrobacterium
Cellulomonas
Arthrobacter
Nocardioides
Cellulosimicrobium
Oerskovia
Sanguibacter
Actinobacteria / Firmicutes (Group 5)
Peptostreptococcus
Deinococcus
5

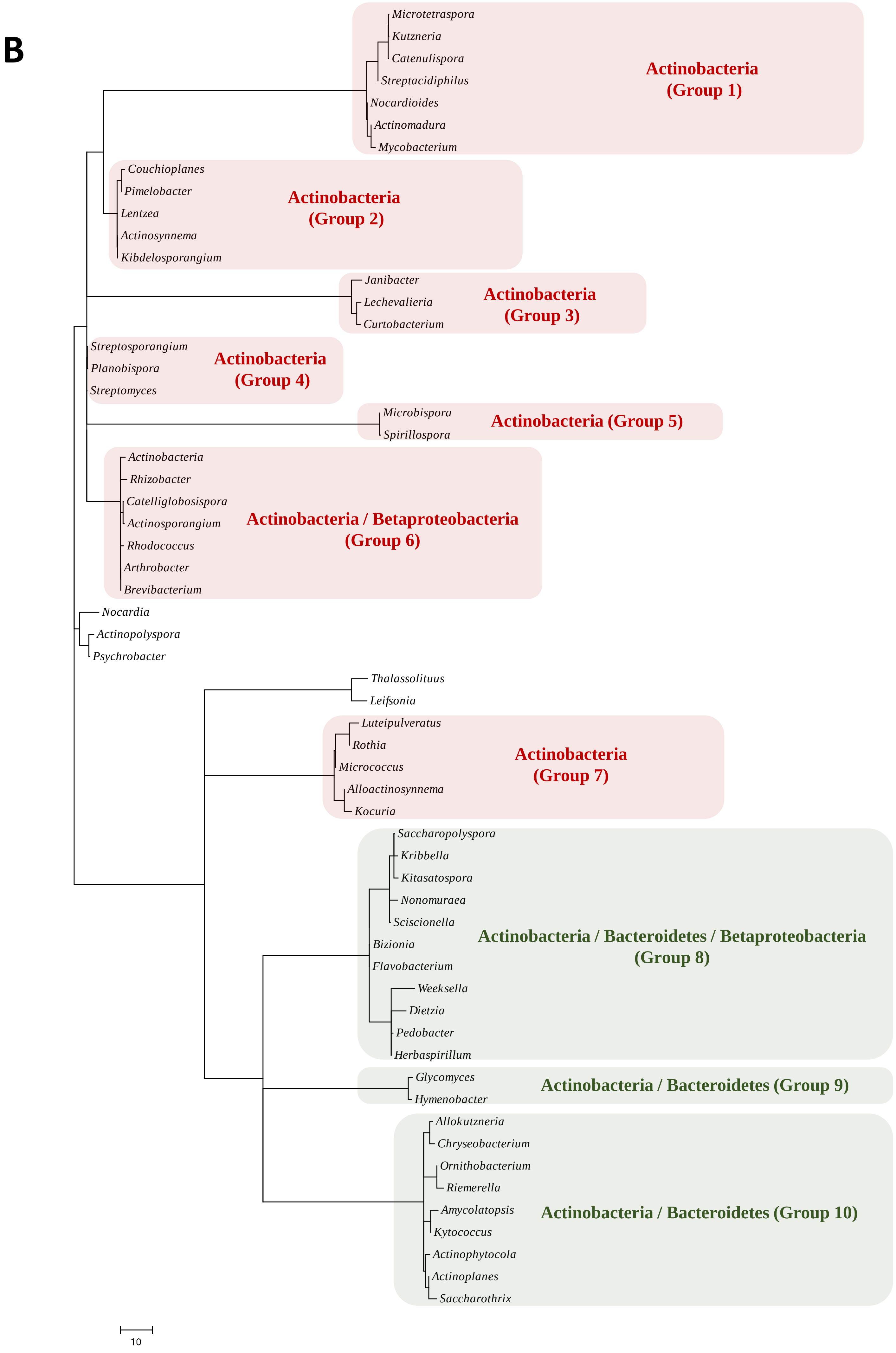

B
Microtetraspora
Kutzneria
Catenulispora
Streptacidiphilus
Nocardioides
Actinomadura
Mycobacterium
Actinobacteria (Group 1)
Couchioplanes
Pimelobacter
Lentzea
Actinosynnema
Kibdelosporangium
Actinobacteria (Group 2)
Janibacter
Lechevalieria
Curtobacterium
Actinobacteria (Group 3)
Streptosporangium
Planobispora
Streptomyces
Actinobacteria (Group 4)
Microbispora
Spirillospora
Actinobacteria (Group 5)
Actinobacteria
Rhizobacter
Catelliglobosispora
Actinosporangium
Rhodococcus
Arthrobacter
Brevibacterium
Actinobacteria / Betaproteobacteria (Group 6)
Nocardia
Actinopolyspora
Psychrobacter
Thalassolituus
Leifsonia
Luteipulveratus
Rothia
Micrococcus
Alloactinosynnema
Kocuria
Actinobacteria (Group 7)
Saccharopolyspora
Kribbella
Kitasatospora
Nonomuraea
Sciscionella
Bizionia
Flavobacterium
Weeksella
Dietzia
Pedobacter
Herbaspirillum
Actinobacteria / Bacteroidetes / Betaproteobacteria (Group 8)
Glycomyces
Hymenobacter
Actinobacteria / Bacteroidetes (Group 9)
Allokutzneria
Chryseobacterium
Ornithobacterium
Riemerella
Amycolatopsis
Kytococcus
Actinophytocola
Actinoplanes
Saccharothrix
Actinobacteria / Bacteroidetes (Group 10)
10

Supplemental Figure 1. A) Phylogenetic tree of bile salt hydrolase sequences retrieved from the NCBI database. The evolutionary history was inferred by using the Maximum Likelihood method based on the Le_Gascuel_2008 model [1]. The tree with the highest log likelihood (-31878.76) is shown. Initial tree(s) for the heuristic search were obtained automatically by applying Neighbor-Join and BioNJ algorithms to a matrix of pairwise distances estimated using a JTT model, and then selecting the topology with superior log likelihood value. A discrete Gamma distribution was used to model evolutionary rate differences among sites (5 categories (+G, parameter = 1.6195)). The rate variation model allowed for some sites to be evolutionarily invariable ([+I], 0.32% sites). The tree is drawn to scale, with branch lengths measured in the number of substitutions per site. The analysis involved 72 amino acid sequences. All positions containing gaps and missing data were eliminated. There were a total of 308 positions in the final dataset. Evolutionary analyses were conducted in MEGA7 [3]. The tree was divided into groups, designed as Group 1 to 5, depending on the grouping at phylum level. **B) Phylogenetic tree of cholesterol oxidase sequences retrieved from the NCBI database**. The evolutionary history was inferred by using the Maximum Likelihood method based on the Whelan And Goldman + Freq. model [2]. The tree with the highest log likelihood (-71531.84) is shown. Initial tree(s) for the heuristic search were obtained automatically by applying Neighbor-Join and BioNJ algorithms to a matrix of pairwise distances estimated using a JTT model, and then selecting the topology with superior log likelihood value. A discrete Gamma distribution was used to model evolutionary rate differences among sites (5 categories (+G, parameter = 14.7797)). The rate variation model allowed for some sites to be evolutionarily invariable ([+I], 0.19% sites). The tree is drawn to scale, with branch lengths measured in the number of substitutions per site. The analysis involved 59 amino acid sequences. All positions containing gaps and missing data were eliminated. There were a total of 515 positions in the final dataset. Evolutionary analyses were conducted in MEGA7 [3]. The tree was divided into groups, designed as Group 1 to 10, depending on the grouping at phylum level.

1. Le SQ, Gascuel O. 2008. An Improved General Amino Acid Replacement Matrix. Mol Biol Evol 25:1307-1320.
2. Whelan S, Goldman N. 2001. A general empirical model of protein evolution derived from multiple protein families using a maximum-likelihood approach. Mol Biol Evol 18:691-699.
3. Kumar S, Stecher G, Tamura K. 2016. MEGA7: Molecular Evolutionary Genetics Analysis version 7.0 for bigger datasets. Mole Biol Evol 33:1870-1874.
